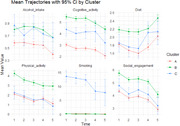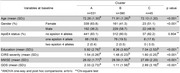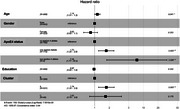# Lifestyle habits pattern and risk of late‐life dementia: findings from a population‐based study

**DOI:** 10.1002/alz70860_105028

**Published:** 2025-12-23

**Authors:** Michele Rossi, Irene Brianzoni, Mauro Colombo, Antonio Guaita, Elena Rolandi

**Affiliations:** ^1^ Golgi Cenci Foundation, Abbiategrasso, Milan, Italy

## Abstract

**Background:**

By 2050, the global population aged 60+ will exceed 2.1 billion, making cognitive health a priority. Previous research highlighted the importance of maintaining a healthy lifestyle for dementia prevention, however to date it is not clear the natural history of multiple health‐related behaviors in older populations. This study aimed to identify distinct trajectories of multiple lifestyle habits and their association with dementia risk in a population‐base sample of older adults followed for 12 years.

**Method:**

The InveCe.Ab study (NCT01345110) recruited individuals aged 70–74 in Abbiategrasso (Milan) at baseline (November 2009–January 2011) and followed them after 2, 4, 8, and 12 years. We include participants without dementia at baseline and performing at least two follow‐ups (*n* = 966). A multivariate longitudinal K‐means clustering approach grouped participants based on the longitudinal patterns of six lifestyle habits (physical activity, healthy diet, smoking, alcohol use, cognitive activity, and social engagement). Cox proportional hazards models examined the risk of incident dementia across clusters, adjusting for age, sex, education, and ApoE4 status.

**Result:**

Over the follow‐up, 159 participants (16.5%) developed dementia. Cluster analysis identified 3 different clusters (Figure 1). Cluster B (*n* = 390), characterized by healthier lifestyle patterns, was slightly younger, more educated, predominantly male (table 1). Compared with cluster B, cluster A (*n* = 531) had a 1.7‐fold higher risk of dementia, while the less prevalent cluster C (*n* = 45) showed a non‐significant increase with a wide confidence interval (table 2).

**Conclusion:**

Among community‐dwelling older adults, 40% displayed a pattern of health‐related behaviors characterized by consistently higher engagement in multiple leisure‐time activities, no smoking, healthy diet, and moderate alcohol consumption. This lifestyle pattern was associated with reduced dementia incidence in late‐life, controlling for non‐modifiable factors. These findings underscore the importance of multifaceted lifestyle habits in preserving cognitive health among older adults and could inform future community‐level preventive initiatives.